# Psychological and Behavioral Pathways from Perceived Teacher Support to School Belonging Among Vocational Students: A Three-Wave Longitudinal Study

**DOI:** 10.3390/bs16081288

**Published:** 2026-07-28

**Authors:** Jiliang Liu, Yunbo Liu, Wenli Wang

**Affiliations:** 1 School of Humanities and Education, Guangzhou Institute of Science and Technology, Guangzhou 510540, China; jlliu@gzist.edu.cn; 2 Faculty of Education, Beijing Normal University, Beijing 100875, China; yunboliu@bnu.edu.cn; 3 Human Resources Department, Xiamen Institute of Technology, Xiamen 361021, China

**Keywords:** perceived teacher support, school belonging, self-efficacy, learning engagement, secondary vocational education, longitudinal study

## Abstract

School belonging is an important indicator of students’ school adjustment and psychological well-being, yet it may be particularly vulnerable among students in Chinese secondary vocational education because of early academic tracking and weak vocational identity. This three-wave longitudinal study examined whether perceived teacher support was associated with later school belonging through self-efficacy and learning engagement. Data were collected from first-year secondary vocational students in China. The baseline valid sample included 9116 students, and 1273 students were retained at Time 3, indicating substantial attrition across waves. Structural equation modeling was used to test the serial mediation model, controlling for baseline self-efficacy, learning engagement, and school belonging. The results showed that perceived teacher support at Time 1 was positively associated with self-efficacy at Time 2, which was associated with learning engagement at Time 2. Learning engagement was positively associated with school belonging at Time 3. The direct association between perceived teacher support and school belonging was also significant but modest, and the serial indirect association through self-efficacy and learning engagement was statistically significant but small in magnitude. These findings contribute to the literature by showing that vocational students’ school belonging is linked not only to perceived teacher–student support, but also to a gradual psychological–behavioral process involving competence beliefs and learning participation.

## 1. Introduction

School belonging is widely recognized as a critical indicator of students’ positive school adjustment and psychological well-being (Pendergast et al., 2020). A growing body of research has demonstrated that a strong sense of school belonging is closely associated with higher academic engagement, learning persistence, and subjective well-being, while also reducing dropout risk and buffering the negative effects of academic stress (Adinata et al., 2025; Fenizia & Parrello, 2025; Nix et al., 2022). Importantly, school belonging is not evenly distributed across student populations. Students positioned at the margins of educational systems are more likely to experience diminished belonging, a challenge that is particularly salient among students in secondary vocational education.

In the Chinese context, secondary vocational education is closely intertwined with the high-stakes tracking system following the junior secondary school entrance examination. A substantial proportion of students enter vocational schools not as a result of deliberate career planning or strong vocational interests, but rather as a consequence of failing to meet academic thresholds for general upper secondary education. Such tracking experiences are often accompanied by self-attributions of academic failure, negative social labeling, and unfavorable perceptions of the social status of vocational education, which collectively undermine students’ initial identification with their schools and fields of study (Xiong, 2022). Consequently, secondary vocational students are more likely to exhibit feelings of alienation and psychological distance during the early stages of schooling, rendering the development of school belonging particularly challenging (National Institute of Vocational and Technical Education, Ministry of Education, 2016).

From a developmental and contextual interaction perspective, school belonging should not be understood as a dynamic psychological experience that is gradually constructed through everyday interactions within the school environment (K. Allen et al., 2018). In vocational education settings, teachers often constitute the most salient and stable social agents in students’ school lives. Their instructional practices and interaction styles play a pivotal role in shaping students’ overall perceptions of school (Ibrahim & El Zaatari, 2020). Previous research suggests that supportive teaching environments enable students to feel noticed, respected, and accepted, thereby laying an essential foundation for the formation of school belonging (Walker & Graham, 2021).

Teacher support extends beyond the clear delivery of instructional content to encompass teachers’ emotional care, competence-related feedback, and learning guidance during the instructional process (Tao et al., 2022). Supportive teaching behaviors are believed to be associated with students’ school adjustment indirectly by activating key psychological resources and motivational processes (Ryan & Deci, 2020; Huang & Wang, 2023). Specifically, when students perceive understanding and encouragement from teachers, they are more likely to develop positive beliefs about their learning capabilities, reflected in enhanced self-efficacy (Guo et al., 2025; Shao et al., 2025). In this study, teacher support refers to students’ perceived teacher support. Elevated self-efficacy, in turn, promotes greater effort, persistence, and involvement in learning activities (Ibrahim & El Zaatari, 2020; Shao et al., 2025). This sequence from competence beliefs to learning participation may provide one pathway through which perceived teacher support is linked to students’ sense of participation and belonging within the school context (Nix et al., 2022).

Despite growing evidence linking teacher support, self-efficacy, and learning engagement to students’ school belonging, several limitations remain in the existing literature. First, the majority of prior studies have relied on cross-sectional designs, which restrict the ability to capture the temporal ordering and process through which teacher support is linked to the development of school belonging over time (Nix et al., 2022). As a result, the dynamic mechanisms underlying this association remain insufficiently understood. Second, empirical research in this area has predominantly focused on general education settings, while comparatively little attention has been paid to students in secondary vocational education, who often face distinct structural and psychosocial challenges. More importantly, few studies have examined longitudinally whether perceived teacher support is linked to school belonging through a serial psychological–behavioral pathway involving both self-efficacy and learning engagement.

To address these gaps, the present study focuses on secondary vocational students in China and employs a three-wave longitudinal design to examine the mediating roles of self-efficacy and learning engagement in the association between teacher support and students’ subsequent sense of school belonging. In this model, self-efficacy represents students’ beliefs about their learning capability, whereas learning engagement reflects their behavioral involvement in learning activities. By arranging these variables in a temporal sequence, the study aimed to clarify whether early perceived teacher support was linked to later school belonging through students’ competence beliefs and learning participation. Given the observational nature of the data, the hypothesized pathways were interpreted as longitudinal associations rather than causal effects.

### Theoretical Framework and Hypothesis Development

Within school contexts, teacher support is widely regarded as a critical external resource shaping students’ learning processes and psychological adjustment. By providing academic guidance, emotional care, and constructive feedback, teachers can satisfy students’ needs for competence and relatedness, thereby influencing how students experience and interpret their school environment (Ibrahim & El Zaatari, 2020). School belonging, defined as students’ emotional attachment to school and their sense of membership, represents a developmental and relatively stable indicator of school adjustment (K.-A. Allen et al., 2024). In vocational education settings in particular, school belonging has been shown to play a pivotal role in students’ learning persistence and vocational identity development. Accordingly, teacher support may provide an important contextual condition associated with later school belonging.

From a social–cognitive and motivational perspective, the influence of teacher support on school belonging may unfold not only through direct relational experiences, but also through students’ internal psychological resources and learning-related behaviors over time (Sun et al., 2025). In prior study, teacher support amplifies students’ interest, enjoyment, and engagement in learning (Wong et al., 2018), while emotional support from teachers has been shown to cultivate positive attitudes and intrinsic motivation, prompting greater effort and commitment to academic tasks (Liu et al., 2023). These forms of support may enhance students’ self-efficacy, thereby strengthening their academic engagement and, in turn, fostering a greater sense of belonging at school.

Self-efficacy reflects students’ beliefs in their capability to successfully accomplish learning tasks. Teachers’ instructional guidance, feedback, and encouragement provide salient competence-related cues that can strengthen students’ self-efficacy across time (Huang & Wang, 2023; Zhao et al., 2023). This process may be particularly salient in vocational education, where students often encounter repeated challenges and setbacks in skill-based learning contexts. Thus, supportive teaching practices are expected to be particularly relevant to vocational students’ self-efficacy. Based on this reasoning, the present study proposes that teacher support at Time 1 positively predicts students’ self-efficacy at Time 2.

Beyond its direct motivational function, self-efficacy is also considered a core psychological mechanism driving students’ learning involvement. Students with higher levels of self-efficacy are more likely to invest effort, persist in the face of difficulties, and actively participate in classroom activities. Sustained engagement in learning activities, in turn, increases opportunities for successful experiences and social interactions, which may gradually enhance students’ identification with and emotional attachment to school (Kern & Wehmeyer, 2021). In this sense, self-efficacy may exert an indirect influence on school belonging by promoting higher levels of learning engagement.

Because school belonging develops through ongoing interactions between students and their school environment, the present study adopts a longitudinal serial mediation framework to examine whether early perceived teacher support is associated with later school belonging through students’ self-efficacy and learning engagement. In this model, perceived teacher support at Time 1 represents students’ early perception of instructional and relational support, self-efficacy and learning engagement at Time 2 represent psychological and behavioral mediating variables, and school belonging at Time 3 represents subsequent school adjustment. The direct path from perceived teacher support to later school belonging was retained as a baseline hypothesis because prior research has consistently shown positive associations between supportive teacher–student relationships and students’ sense of belonging. At the same time, the theoretical emphasis of this study lies in the indirect psychological–behavioral pathway, which may better explain how perceived teacher support is linked to later belonging among vocational students. Thus, the direct and indirect hypotheses are complementary rather than contradictory.

Based on the theoretical framework outlined above, the following hypotheses are proposed:

**H1.**  *Perceived teacher support at Time 1 is positively associated with school belonging at Time 3.*

**H2.**  *Perceived teacher support at Time 1 is positively associated with self-efficacy at Time 2.*

**H3.**  *Self-efficacy at Time 2 is positively associated with learning engagement at Time 2.*

**H4.**  *Self-efficacy and learning engagement at Time 2 jointly mediate the longitudinal association between perceived teacher support at Time 1 and school belonging at Time 3 in a serial manner.*

## 2. Materials and Methods

### 2.1. Participants

The present study drew on data from three waves of the China Vocational Education Student Development Survey conducted by the National Institute of Vocational Education, Beijing Normal University. Data were collected in December 2022 (Time 1), June 2023 (Time 2), and December 2024 (Time 3). The baseline survey covered secondary vocational schools across eastern, central, and western regions of China, including Beijing, Jiangsu, Henan, Inner Mongolia, and Sichuan. The second and third waves followed up the baseline participants. Participants were matched across waves using anonymous student identification codes generated during school-based survey administration. These codes were used only for longitudinal matching, and personally identifiable information was not included in the analytic dataset.

A stratified proportional sampling strategy was adopted. Within each province, secondary vocational schools were selected based on school ownership (public vs. private), student enrollment size, and geographic location. Within each sampled school, two to three majors were selected from typical disciplinary clusters, such as equipment manufacturing, electronics and information technology, and medical and health services. These major clusters were selected because they represented common program areas in the participating vocational schools and allowed the sample to include both technical and service-oriented fields. For each major, one to two administrative classes were randomly selected from each grade level, and students completed the questionnaire online in a classroom setting.

Given the longitudinal focus on students’ development during their first three years of vocational schooling, the present study restricted the analytic sample to students who were in their first year of secondary vocational education at the Time 1 survey. At Time 1, a total of 9960 questionnaires were returned, of which 9116 were valid, yielding a valid response rate of 93.14%. At Time 2, 4752 students were successfully followed up from the baseline sample, corresponding to a retention rate of 50.35%. At Time 3, valid data were obtained from 1273 students, representing an overall retention rate of 13.9% relative to the Time 1 valid sample. The final analytic sample therefore consisted of 1273 first-year baseline students who were successfully matched across the three waves. Among them, 746 were male students (58.6%) and 527 were female students (41.4%). Because the analytic sample was restricted to the same entry cohort, all students were in the same grade cohort at baseline.

All surveys were administered during regular class time under the guidance of trained research assistants. Participation was voluntary, and written informed consent was obtained from all students and their legal guardians prior to data collection. The study protocol was reviewed and approved by the Ethics Committee of Beijing Normal University (Approval No. BNU202210100036).

### 2.2. Attrition Analysis

Given the multi-wave longitudinal design of this study, an attrition analysis was conducted to examine whether sample loss introduced systematic bias. Specifically, students who completed all three waves of the survey were compared with those who dropped out at later waves on baseline (Time 1) demographic characteristics and key study variables.

Chi-square analysis indicated that retention status was not significantly associated with gender distribution, χ^2^(1) = 0.36, *p* = 0.548, suggesting that attrition was not systematically related to gender. Independent-samples t tests further showed that students who completed all three waves reported significantly higher baseline levels of teacher support and self-efficacy than those who dropped out (*p*s < 0.001), whereas no significant difference was observed for learning engagement (*p* > 0.05). Although some baseline differences reached statistical significance, the corresponding effect sizes were small (Cohen’s d ≈ 0.20). According to conventional benchmarks (Cohen, 1988), these differences fall within the range of small effects. Prior methodological research has also noted that small effect sizes are common in psychological and educational research, particularly in large-sample and longitudinal studies, and that statistical significance does not necessarily imply substantial practical differences (Funder & Ozer, 2019; Gignac & Szodorai, 2016).

Nevertheless, the overall attrition rate was substantial, with only 13.9% of the baseline valid sample retained at Time 3. Therefore, the possibility of selection bias cannot be ruled out. The attrition analyses suggest that retained and non-retained students were broadly comparable on some baseline characteristics, but they do not fully eliminate concerns about external validity or the representativeness of the final longitudinal sample. In subsequent longitudinal structural equation models, baseline levels of the relevant variables were controlled to reduce the potential influence of initial group differences. Missing data were handled using full information maximum likelihood (FIML) estimation under the missing-at-random assumption. However, because the MAR assumption cannot be fully verified with the available data, the findings should be interpreted as applying most directly to students who remained trackable across the three waves.

### 2.3. Instruments

#### 2.3.1. Perceived Teacher Support

Perceived teacher support was conceptualized as students’ perceptions of teachers’ instructional and relational support rather than directly observed teacher behavior. It comprised two related but distinct dimensions: teacher–student interaction and teacher instructional enthusiasm. Teacher–student interaction was assessed using five items adapted from the Effective Graduate Survey developed by Wu and Guo (2019), such as “Teachers emphasize guidance and inspiration to stimulate students’ interest in learning.” Teacher instructional enthusiasm was measured with three items drawn from the Teacher Enthusiasm Scale in the PISA 2018 student questionnaire (OECD, 2019), including items such as “I can clearly feel that my teachers enjoy teaching us.” All items were rated on a 4-point Likert scale (1 = strongly disagree, 4 = strongly agree).

Consistent with the research framework, perceived teacher support was measured at Time 1 and Time 3. In this study, the two dimensions were specified as separate but correlated latent factors. Confirmatory factor analysis (CFA) supported this two-factor structure at both measurement occasions. All items loaded significantly on their intended latent constructs, with standardized factor loadings exceeding acceptable thresholds (minimum standardized loading = 0.740), indicating satisfactory convergent validity and measurement stability across time. Internal consistency was acceptable at both waves, with Cronbach’s α values of 0.963 at T1 and 0.90 at T3.

#### 2.3.2. Self-Efficacy

Self-efficacy was measured using five items adapted from the self-efficacy/resilience scale (ST188) in PISA 2018 (OECD, 2019). A sample item is “I usually find ways to achieve my goals.” Responses were recorded on a 5-point Likert scale (1 = strongly disagree, 5 = strongly agree). Self-efficacy was assessed at Time 1, Time 2, and Time 3, although the main mediation model used Time 1 as the baseline control and Time 2 as the mediator.

CFA results indicated that all items demonstrated adequate standardized loadings on the corresponding latent factor at both time points, with the lowest standardized factor loading equal to 0.718. These results suggest that the self-efficacy measure exhibited good convergent validity and temporal stability. Internal consistency was acceptable across waves, with Cronbach’s α values ranging from 0.921 to 0.939.

#### 2.3.3. Learning Engagement

Learning engagement was assessed using three items from the Chinese version of the Learning Engagement Scale revised by Fang et al. (2008). These items capture students’ concentration and absorption in learning activities. A sample item is “When I am studying, I often forget everything around me.” Items were rated on a 5-point Likert scale (1 = not at all like me, 5 = very much like me). Although the broader survey included additional items related to reduced academic accomplishment, these items reflected academic burnout rather than learning engagement. Therefore, only the three engagement items were used to represent learning engagement in the present study. Learning engagement was assessed at all three waves. In the main longitudinal model, Time 1 learning engagement was used as a baseline control, and Time 2 learning engagement was specified as the behavioral mediator.

The three-item learning engagement measure showed acceptable internal consistency at Time 2 and Time 3, with Cronbach’s α values of 0.705 and 0.900, respectively, although internal consistency was relatively low at Time 1 (α = 0.575). CFA results indicated that all three items loaded strongly on the latent construct across the waves used in the main model, with the lowest standardized loading equal to 0.853. Because Time 1 learning engagement was used only as a baseline control, this reliability limitation was considered when interpreting the findings.

#### 2.3.4. School Belonging

School belonging was measured using five items from the PISA 2018 student questionnaire (ST034; OECD, 2019). The items assess students’ feelings of inclusion and connectedness at school, with statements such as “I feel like an outsider at school.” All items were rated on a 5-point Likert scale (1 = not at all like me, 5 = very much like me). School belonging was assessed at Time 2 and Time 3 in the main longitudinal model, with Time 2 school belonging used as the baseline control and Time 3 school belonging specified as the outcome variable.

CFA results demonstrated that all items loaded strongly on the school belonging latent factor at each measurement occasion, with all standardized factor loadings equal to or above 0.846. These findings indicate acceptable convergent validity for the school belonging scale. Cronbach’s α was 0.639 at Time 2 and 0.849 at Time 3, indicating modest internal consistency at Time 2 and good internal consistency at Time 3.

### 2.4. Analysis Plan

A longitudinal structural equation modeling (SEM) approach was employed to examine the longitudinal associations among perceived teacher support, self-efficacy, learning engagement, and school belonging, as well as the serial mediating roles of self-efficacy and learning engagement. Because the study used observational survey data, all structural paths were interpreted as longitudinal associations rather than causal effects. All analyses were conducted using SPSS 29.0 for preliminary analyses and AMOS 29.0 for confirmatory factor analyses and structural equation modeling.

First, descriptive statistics (means and standard deviations) and Pearson correlation coefficients were computed for all study variables across measurement waves to provide an initial overview of variable distributions and bivariate associations. Next, confirmatory factor analyses (CFAs) were conducted to evaluate the measurement models of teacher support, self-efficacy, learning engagement, and school belonging at each wave. To address the comparability of repeated measures across time, longitudinal measurement invariance was tested sequentially for constructs measured at multiple waves. Configural, metric, and scalar invariance models were estimated, and changes in model fit were evaluated using ∆CFI, and ∆RMSEA. Measurement invariance was considered supported when decreases in CFI were no greater than 0.010 and changes in RMSEA remained within commonly recommended thresholds.

After establishing acceptable measurement model fit, longitudinal structural models were specified to test the hypothesized relationships among the variables. Perceived teacher support at Time 1 was modeled as the exogenous predictor, self-efficacy and learning engagement at Time 2 as serial mediators, and school belonging at Time 3 as the outcome variable. Baseline levels of self-efficacy, learning engagement, and school belonging at the preceding time points were controlled where available, including self-efficacy at Time 1, learning engagement at Time 1, and school belonging at Time 2. Perceived teacher support at Time 3 was not included as a control variable in the mediation model because it was measured concurrently with school belonging at Time 3. This decision was made to avoid controlling for a same-wave construct that overlaps conceptually with the outcome.

Model fit was evaluated using multiple indices, including the comparative fit index (CFI), Tucker–Lewis index (TLI), root mean square error of approximation (RMSEA), and standardized root mean square residual (SRMR). Values of CFI and TLI ≥ 0.90 and RMSEA and SRMR ≤ 0.08 were considered indicative of acceptable model fit. Because CFI and TLI values closer to 0.95 are often regarded as evidence of stronger fit in SEM research, model evaluation was based on the overall pattern of fit indices rather than on a single cutoff.

The longitudinal serial mediation effects of teacher support on school belonging were tested using bias-corrected bootstrap procedures with 5000 resamples to estimate 95% confidence intervals for indirect effects. Indirect associations were considered significant when the confidence interval did not include zero. Both statistical significance and effect magnitude were considered when interpreting mediation results.

To examine the robustness of the findings, supplementary analyses were conducted by including demographic variables, such as gender, grade level, and family socioeconomic status, as covariates in the structural model. Path coefficients obtained from models with and without covariates were compared to assess the stability of the main findings. Statistical significance was evaluated at the 0.05 level.

## 3. Results

### 3.1. Descriptive Statistics and Correlation Analyses

Table 1 presents the means, standard deviations, and Pearson correlation coefficients for the study variables across measurement waves. Overall, the means were within reasonable ranges, and the standard deviations indicated sufficient interindividual variability, providing an appropriate basis for subsequent structural equation modeling analyses.

At each measurement wave, learning engagement, self-efficacy, teacher support, and school belonging were generally positively correlated with one another. For example, at Time 2, learning engagement was significantly and positively associated with teacher support (r = 0.293, *p* < 0.01), self-efficacy (r = 0.528, *p* < 0.01), and school belonging (r = 0.199, *p* < 0.01). Similar patterns of moderate to strong positive correlations were observed among the core variables at Time 3 (rs = 0.593–0.681, *p*s < 0.01), suggesting a high degree of structural coherence among these constructs within the same measurement occasions.

Longitudinally, perceived teacher support at Time 1 showed modest positive associations with later psychological and behavioral outcomes. Specifically, teacher support at Time 1 was positively correlated with self-efficacy at Time 2 (r = 0.297, *p* < 0.01), as well as with learning engagement at Time 3 (r = 0.228, *p* < 0.01) and school belonging (r = 0.171, *p* < 0.01) at Time 3. These findings suggest that teacher support may exert a sustained influence on students’ subsequent development over time. The correlations among repeated measures and later outcomes varied in magnitude, suggesting that the longitudinal associations were generally modest.

Self-efficacy also showed positive associations with both concurrent and later learning-related variables. For example, self-efficacy at Time 2 was positively correlated with learning engagement at Time 2 (r = 0.439, *p* < 0.01) and school belonging at Time 3 (r = 0.259, *p* < 0.01). These bivariate associations were consistent with the proposed longitudinal model, although they do not establish mediation by themselves. The correlational results were broadly consistent with the proposed longitudinal serial mediation model, while also showing that some zero-order associations were modest. These descriptive findings provided a preliminary basis for the subsequent measurement model evaluation and structural model testing.

### 3.2. Measurement Model Evaluation and Longitudinal Measurement Invariance

Prior to testing the structural relationships, longitudinal CFA models were estimated for the four repeated constructs. As shown in Table 2, configural models generally showed acceptable fit, indicating that the same factor structures were tenable across waves. The perceived teacher support model showed acceptable CFI and TLI values, although its RMSEA was slightly above the conventional 0.08 threshold; therefore, this model was interpreted with caution.

Longitudinal measurement invariance was further examined by comparing configural, metric, and scalar models. Metric invariance was supported for all constructs, as changes in CFI and RMSEA from configural to metric models were within recommended thresholds. These results indicate that factor loadings were comparable across time, supporting the interpretation of longitudinal associations among the latent constructs. Scalar invariance was not consistently supported, particularly for school belonging, perceived teacher support, and learning engagement. Scalar invariance is primarily required for comparing latent means across time, whereas metric invariance provides a basis for comparing structural associations among latent constructs (Vandenberg & Lance, 2000; Widaman et al., 2010). Because the present study focused on longitudinal structural associations rather than latent mean changes, the lack of full scalar invariance did not preclude testing the hypothesized SEM paths, although it remains a measurement limitation. Therefore, the structural findings should be interpreted as associations among latent constructs rather than as evidence of changes in construct levels over time.

### 3.3. Structural Model Testing

After establishing acceptable measurement models, a longitudinal serial mediation model was specified to examine whether perceived teacher support at Time 1 was associated with school belonging at Time 3 directly and indirectly through self-efficacy and learning engagement at Time 2. The model controlled for baseline levels of self-efficacy, learning engagement, and school belonging, including self-efficacy at Time 1, learning engagement at Time 1, and school belonging at Time 2. Perceived teacher support at Time 3 was not included as a control variable because it was measured concurrently with school belonging at Time 3.

The serial mediation model (see Table 3) showed acceptable fit to the data, χ^2^ = 1989.160, df = 332, χ^2^/df = 5.991, CFI = 0.946, TLI = 0.939, and RMSEA = 0.063, 90% CI [0.060, 0.065]. As shown in Table 3, perceived teacher support at Time 1 was positively associated with self-efficacy at Time 2 (*β* = 0.145, *p* < 0.001). Self-efficacy at Time 2 was positively associated with learning engagement at Time 2 (*β* = 0.606, *p* < 0.001), and learning engagement at Time 2 was positively associated with school belonging at Time 3 (*β* = 0.121, *p* < 0.001). The direct path from perceived teacher support at Time 1 to school belonging at Time 3 was also statistically significant but modest (*β* = 0.069, *p* = 0.019).

The autoregressive paths were also statistically significant. Self-efficacy at Time 1 was positively associated with self-efficacy at Time 2 (β = 0.381, *p* < 0.001), and learning engagement at Time 1 was positively associated with learning engagement at Time 2 (β = 0.266, *p* < 0.001). School belonging at Time 2 was negatively associated with school belonging at Time 3 after the other variables were controlled (β = −0.121, *p* < 0.001). This negative coefficient should be interpreted as a conditional association in the fully adjusted model rather than as evidence that higher earlier belonging generally led to lower later belonging.

**Figure 1:**
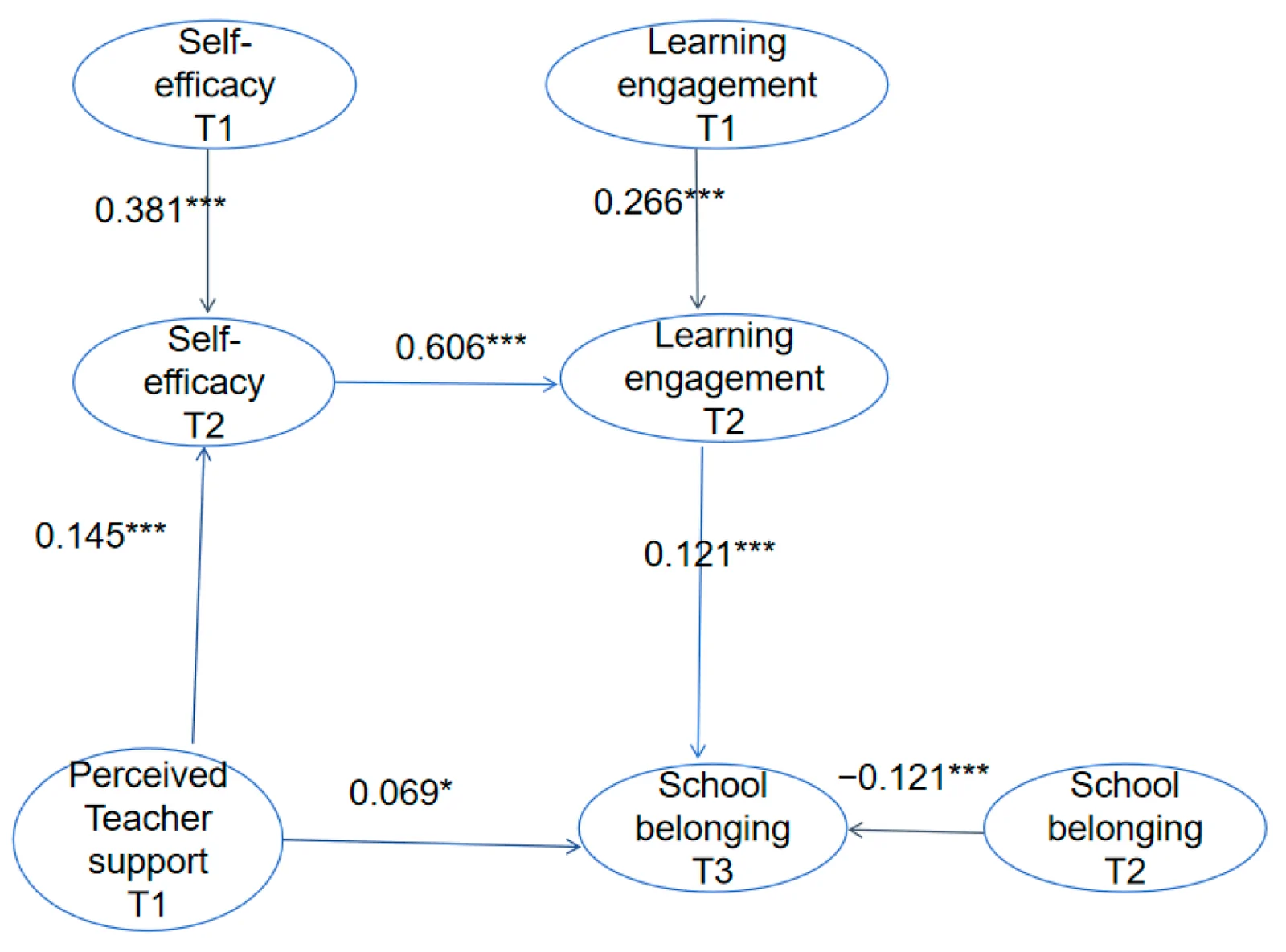
Longitudinal serial mediation model linking perceived teacher support to school belonging through self-efficacy and learning engagement. Standardized path coefficients are reported. Solid lines indicate statistically significant paths. The model controlled for baseline self-efficacy, baseline learning engagement, and prior school belonging. Measurement indicators and residual correlations are omitted for visual clarity. * *p* < 0.05, *** *p* < 0.001.

The serial mediation structural model is shown in Figure 1.

To further examine the indirect effects, bias-corrected bootstrap analyses with 5000 resamples were conducted to estimate the magnitude and significance of the mediation pathways (see Table 4). The direct association between perceived teacher support at Time 1 and school belonging at Time 3 was statistically significant (B = 0.079, β = 0.069, 95% bootstrap CI [0.010, 0.154]). The serial indirect association through self-efficacy at Time 2 and learning engagement at Time 2 was also significant (B = 0.012, β = 0.011, 95% bootstrap CI [0.005, 0.023]). The total association was significant as well (B = 0.091, β = 0.080, 95% bootstrap CI [0.022, 0.167]). These results indicate a partial mediation pattern, although the indirect association was small in magnitude. This modest indirect association is understandable in a multi-wave SEM model that includes baseline controls and should be interpreted as a modest longitudinal pathway rather than as a large practical effect.

Overall, perceived teacher support at Time 1 was associated with school belonging at Time 3 both directly and indirectly through self-efficacy and learning engagement. The significant direct and serial indirect associations support a partial mediation pattern, although the indirect pathway was small in magnitude.

## 4. Discussion

Using three-wave longitudinal data, the present study examined the longitudinal associations among perceived teacher support, self-efficacy, learning engagement, and school belonging among secondary vocational students. The results showed that perceived teacher support was modestly associated with later school belonging both directly and indirectly through self-efficacy and learning engagement. These findings support a partial mediation pattern and suggest that school belonging among vocational students may be linked to both relational and psychological–behavioral processes.

### 4.1. Direct and Indirect Associations Between Perceived Teacher Support and School Belonging

Consistent with Hypothesis 1, perceived teacher support at Time 1 was positively associated with school belonging at Time 3. This finding is consistent with prior studies showing that supportive teacher–student relationships are related to stronger school belonging and better school adjustment (Ibrahim & El Zaatari, 2020; Nix et al., 2022). It suggests that students who feel supported by teachers may be more likely to feel accepted and valued at school. For secondary vocational students, this relational support may be especially important because many students enter vocational schools with relatively weak school identification.

However, the direct association was modest in size. This result suggests that perceived teacher support should not be understood as a strong standalone explanation for school belonging. School belonging is likely shaped by multiple factors, including peer relationships, school climate, learning experiences, and students’ own motivational beliefs. This interpretation is in line with the view that school belonging develops through ongoing interactions between students and their school environment (K. Allen et al., 2018). Therefore, teacher support should be understood as one relational condition associated with belonging, rather than as the only or dominant source of belonging.

The mediation results further showed that perceived teacher support was indirectly associated with school belonging through self-efficacy and learning engagement. This finding is consistent with motivational perspectives suggesting that teacher support may strengthen students’ competence beliefs and support greater involvement in learning (Ryan & Deci, 2020; Huang & Wang, 2023). Rather than linking teacher support and belonging through a single direct pathway, the present results suggest that students’ confidence in learning and their participation in learning activities may help explain how perceived teacher support is connected to later school belonging.

This indirect pathway is particularly relevant in the context of Chinese secondary vocational education. Many vocational students enter vocational schools after early academic tracking and may carry negative experiences of academic failure or social labeling (National Institute of Vocational and Technical Education, Ministry of Education, 2016; Xiong, 2022). Under these conditions, students’ emotional connection to school may not develop immediately in response to teacher support alone. Instead, perceived teacher support may first be associated with students’ confidence in their learning capability. Stronger self-efficacy may then encourage students to participate more actively in learning activities. Through repeated participation and successful learning experiences, students may gradually develop a stronger sense of identification with school.

### 4.2. The Role of Self-Efficacy in Linking Perceived Teacher Support and Learning Engagement

The present findings suggest that self-efficacy may serve as one psychological link through which perceived teacher support was positively associated with students’ learning behaviors. Perceived teacher support at Time 1 was positively associated with students’ self-efficacy, which in turn was strongly associated with learning engagement. This pattern is consistent with previous studies showing that teacher support is closely related to students’ academic self-efficacy and engagement (Huang & Wang, 2023; Liu et al., 2023; Shao et al., 2025).

This finding is relevant for secondary vocational students. Many vocational students have experienced academic setbacks before entering vocational schools. These experiences may weaken their confidence in learning and reduce their willingness to participate in classroom activities (Xiong, 2022). In this context, perceived teacher support may provide students with competence-related feedback and emotional encouragement. Such support may help students strengthen their belief that they can complete learning tasks successfully.

Self-efficacy may then become an important psychological condition for learning engagement. Students with stronger self-efficacy are more likely to invest effort, persist when facing difficulties, and participate in learning activities. This may be especially important in vocational education, where students often need to acquire practical skills through repeated practice and correction. When students believe that improvement is possible, they may be more willing to remain involved in both classroom and practical learning tasks.

Therefore, the present findings suggest that perceived teacher support is linked to learning engagement partly through students’ competence beliefs. This result supports the view that psychological beliefs form one possible bridge between educational contexts and students’ learning behaviors. It also indicates that understanding learning engagement among vocational students may require attention not only to instructional design, but also to students’ confidence in their own learning capability.

### 4.3. Learning Engagement as a Behavioral Pathway to School Belonging

The findings further indicate that learning engagement is positively associated with school belonging, although with a relatively modest effect size. This indicates that students’ engagement in learning activities is not solely an academic behavior. It is also related to their emotional connection to the school and their sense of membership within it.

During the vocational education stage, school belonging is often constructed through students’ everyday learning experiences and social interactions within the school environment. Higher levels of learning engagement imply more frequent participation in classroom and practical activities, which increases opportunities for interaction with teachers and peers. Through such interactions, students may gradually develop familiarity with the school environment, a sense of security, and feelings of acceptance and recognition, which may contribute to later school belonging. This finding aligns with prior syntheses highlighting the role of engagement-related processes in the development of school belonging (Nix et al., 2022).

While the pathway from learning engagement to school belonging exhibited a relatively small effect size, its direction remained consistent and statistically significant even after controlling for multiple baseline variables. This means that learning engagement appears to represent one behavioral pathway associated with later school belonging. In longitudinal, small associations may still have cumulative relevance when supported consistently over time, but they should not be overstated as a strong developmental effects. This is particularly relevant for student groups who are vulnerable to disengagement and marginalization.

### 4.4. Theoretical Implications of the Partial Mediation Pattern

The partial mediation pattern offers a more nuanced understanding of how perceived teacher support is related to school belonging. It suggests that perceived teacher support should not be conceptualized only as a direct relational resource, nor only as an indirect motivational resource. Instead, it may be link to school belonging through both channels. On the one hand, supportive teacher–student interactions may directly strengthen students’ feeling of being recognized and accepted. On the other hand, such support may also help students develop stronger competence beliefs and become more engaged in learning. This dual role adds to existing research that has often examined teacher support, motivation, and belonging as separate associations.

This interpretation is particularly relevant for secondary vocational education. For many vocational students, school belonging may not be formed immediately after enrollment. It may require a process of rebuilding confidence and reconstructing school experiences. Early academic tracking and negative social labeling may weaken students’ initial identification with school and learning. In this context, teacher support may matter because it provides students with opportunities to reinterpret their learning capability and school experience. Belonging, therefore, may be understood not only as an emotional response to being cared for, but also a developmental experience related to becoming more confident and more involved in school life.

The findings also support a process-oriented view of school belonging. School belonging is often discussed as an emotional or relational outcome, but the present model highlights its motivational and behavioral foundations. Students may come to feel that they belong not simply because they are told they are valued, but because they gradually experience themselves as capable learners and active participants. This perspective is consistent with theories that emphasize the interaction between contextual support, psychological resources, and behavioral engagement. It also suggests that school belonging may be better understood as a developing experience rather than as a static attitude.

At the same time, the small indirect association indicates that the psychological–behavioral pathway is only one part of a broader developmental system. Self-efficacy and learning engagement help explain part of the association between perceived teacher support and school belonging, but they do not exhaust the mechanism. Other factors, such as peer relationships, school climate, vocational identity, family support, and perceived social status of vocational education, may also shape students’ belonging. Therefore, future theoretical models should move beyond single-path explanations and examine how multiple ecological resources jointly support vocational students’ school adjustment.

### 4.5. Practical Implications

The findings of this study offer several practical implications for practice in vocational education. Teachers may support students’ self-efficacy through clear goal setting, constructive feedback, and emotional support, rather than focusing exclusively on content delivery. Strengthening students’ beliefs in their learning capabilities may serve as one possible starting point for promoting sustained engagement.

Furthermore, schools may design curricula and instructional activities that provide students with more opportunities to actively participate in learning. Structuring classroom and practical learning experiences in ways that encourage involvement and persistence can help translate enhanced self-efficacy into greater behavioral engagement.

Finally, promoting school belonging among vocational students should be understood as a gradual and cumulative process. The small indirect association identified in this study suggests that perceived teacher support, self-efficacy, and learning engagement are unlikely to produce immediate or large changes in school belonging on their own. However, small improvements in students’ competence beliefs and learning participation may still be relevant when they are supported consistently over time. For vocational schools, promoting belonging may require sustained support across teaching, curriculum design, student guidance, and school climate development.

### 4.6. Limitations and Future Directions

Several limitations should be acknowledged. First, the attrition rate across the three waves was substantial. Although attrition analyses showed only small baseline differences between retained and non-retained students, selection bias cannot be ruled out. Therefore, the findings should be generalized with caution and interpreted as most applicable to students who remained trackable across the three waves.

Second, although a stratified proportional sampling strategy was used, the participating schools were not selected through a fully random or nationally representative sampling procedure. The schools were drawn from selected provinces and program areas. Therefore, the findings should be generalized cautiously to the broader population of secondary vocational students in China.

Third, all core variables were measured using student self-report questionnaires. This may introduce common method bias, especially because students with more positive school experiences may report higher perceived teacher support, self-efficacy, engagement, and belonging. In addition, perceived teacher support was measured as students’ perception of support rather than directly observed teacher behavior. Because this study was based on a large-scale longitudinal survey, teacher reports, classroom observations, and behavioral indicators were not available in the analytic dataset. Future studies could incorporate teacher reports, classroom observations, or behavioral indicators to strengthen measurement validity.

Fourth, although configural and metric measurement invariance were generally supported, scalar invariance was not consistently established across all constructs. Because this study focused on longitudinal structural associations rather than latent mean comparisons, this limitation does not directly undermine the main path analyses. Nevertheless, future research should further refine the measures and examine their longitudinal stability in vocational student samples.

Finally, this study used an observational longitudinal design. The temporal ordering of variables supports the examination of longitudinal associations, but causal conclusions cannot be drawn. Reciprocal processes are also possible; for example, students with stronger engagement or belonging may later perceive greater teacher support. Future studies could use cross-lagged panel models, intervention designs, or classroom-level longitudinal data to examine these possibilities more directly.

## 5. Conclusions

Using three-wave longitudinal data from Chinese secondary vocational students, this study examined the longitudinal associations among perceived teacher support, self-efficacy, learning engagement, and school belonging. The results showed that perceived teacher support at Time 1 was modestly associated with school belonging at Time 3. A serial psychological–behavioral pathway through self-efficacy and learning engagement was also observed. These findings suggest that vocational students’ school belonging may be related not only to their perceptions of supportive teacher–student relationships, but also to their confidence in learning and active participation in school activities. By highlighting both relational and psychological–behavioral processes, this study offers evidence for understanding factors associated with school belonging among secondary vocational students.

## Figures and Tables

**Table 1:** Correlation Coefficient Matrix.

Variables	LE-T1	TS-T1	SE-T1	SE-T2	BEL-T2	LE-T2	LE-T3	TS-T3	SE-T3	BEL-T3
1	1	0.293 **	0.528 **	0.199 **	0.029	0.111 **	0.144 **	0.120 **	0.112 **	0.094 **
2		1	0.444 **	0.297 **	−0.087 **	0.024	0.228 **	0.230 **	0.204 **	0.171 **
3			1	0.413 **	0.036	0.154 **	0.265 **	0.213 **	0.279 **	0.224 **
4				1	0.207 **	0.439 **	0.258 **	0.236 **	0.286 **	0.259 **
5					1	0.545 **	0.092 **	0.031	0.015	0.026
6						1	0.164 **	0.082 **	0.136 **	0.107 **
7							1	0.681 **	0.675 **	0.613 **
8								1	0.615 **	0.593 **
9									1	0.675 **
10										1
M ± SD	2.81 ± 0.721	3.37 ± 0.608	3.358 ± 0.823	3.86 ± 0.882	2.37 ± 0.639	2.97 ± 0.777	3.45 ± 0.725	3.58 ± 0.591	4.02 ± 0.816	3.41 ± 0.682

Note. SE = Self-efficacy; TS = Teacher support; LE = Learning engagement; BEL = School Belonging; T1 = Time 1; T2 = Time 2; T3 = Time 3; **, *p* < 0.01.

**Table 2:** Longitudinal CFA results for the measurement models.

Construct	Model	χ^2^	df	CFI	TLI	RMSEA	ΔCFI	ΔRMSEA	Decision
SE	Configural	242.004	29	0.98	0.969	0.076	—	—	Supported
SE	Metric	249.509	33	0.98	0.973	0.072	0	0.004	Supported
SE	Scalar	388.89	38	0.967	0.961	0.085	0.013	0.013	Marginal
BEL	Configural	3.635	5	1	1.001	0	—	—	Supported
BEL	Metric	8.627	7	1	0.999	0.014	0	0.014	Supported
BEL	Scalar	781.58	10	0.85	0.775	0.246	0.15	0.232	Not supported
TS	Configural	555.371	47	0.963	0.948	0.092	—	—	Supported with caution
TS	Metric	582.339	52	0.961	0.951	0.09	0.002	0.002	Supported
TS	Scalar	1579.842	58	0.889	0.873	0.144	0.072	0.054	Not supported
LE	Configural	29.096	5	0.995	0.986	0.062	—	—	Supported
LE	Metric	44.926	7	0.992	0.984	0.065	0.003	0.003	Supported
LE	Scalar	141.995	10	0.974	0.961	0.102	0.018	0.037	Not supported

Note. Configural = same factor structure across waves; Metric = factor loadings constrained equal across waves; Scalar = factor loadings and item intercepts constrained equal across waves. ΔCFI and ΔRMSEA indicate absolute changes relative to the preceding model. Metric invariance was considered supported when ΔCFI ≤ 0.010 and ΔRMSEA ≤ 0.015. Scalar invariance was interpreted cautiously because scalar constraints were not consistently supported across constructs. SE = self-efficacy; BEL = school belonging; TS = perceived teacher support; LE = learning engagement.

**Table 3:** Structural path estimates of the serial mediation model.

Path	B	*β*	SE	CR	*p*
SE_T2 ← TS_T1	0.194	0.145	0.036	5.326	<0.001
SE_T2 ← SE_T1	0.410	0.381	0.031	13.191	<0.001
LE_T2 ← SE_T2	0.659	0.606	0.03	22.211	<0.001
LE_T2 ← LE_T1	0.290	0.266	0.028	10.473	<0.001
BEL_T3 ← BEL_T2	−0.096	−0.121	0.024	−3.983	<0.001
BEL_T3 ← LE_T2	0.094	0.121	0.024	3.94	<0.001
BEL_T3 ← TS_T1	0.079	0.069	0.034	2.339	0.019

Note. TS = perceived teacher support; SE = self-efficacy; LE = learning engagement; BEL = school belonging; T1 = Time 1; T2 = Time 2; T3 = Time 3. B values are unstandardized estimates; β values are standardized estimates; SE values refer to the standard errors of unstandardized estimates.

**Table 4:** Bootstrap test results for serial mediated effects.

Effect	Pathway	B	*β*	95% BC CI for B
Direct effect	TS_T1 → BEL_T3	0.079	0.069	[0.010, 0.154]
Serial indirect effect	TS_T1 → SE_T2 → LE_T2 → BEL_T3	0.012	0.011	[0.005, 0.023]
Total effect	TS_T1 → BEL_T3	0.091	0.08	[0.022, 0.167]

Note: TS = perceived teacher support; SE = self-efficacy; LE = learning engagement; BEL = school belonging. T1 = Time 1; T2 = Time 2; T3 = Time 3. B values are unstandardized estimates; β values are standardized estimates. BC CI = bias-corrected bootstrap confidence interval based on 5000 bootstrap samples.

## Data Availability

The data presented in this study are not publicly available due to privacy and ethical restrictions related to the participation of secondary vocational students. Anonymized data may be made available from the corresponding author upon reasonable request and subject to approval by the relevant ethics committee and data management requirements.
